# (5,10,15,20-Tetra­phenyl­porphyrinato-κ^4^ *N*)(2,2,2-trifluoro-1-phenyl­ethyl­idene-κ*C* ^1^)ruthenium(II): a stable fluorinated alkyl­idene complex of a ruthenium(II) porphyrin

**DOI:** 10.1107/S1600536808023374

**Published:** 2008-08-06

**Authors:** Hidetaka Yuge, Natsuko Arakawa, Satoko Wada, Takeshi Ken Miyamoto

**Affiliations:** a Department of Chemistry, School of Science, Kitasato University, Kitasato, Sagamihara, Kanagawa 228-8555, Japan

## Abstract

In the title compound, [Ru(C_44_H_28_N_4_)(C_8_H_5_F_3_)], the fluorin­ated alkyl­idene group is bound to a five-coordinate Ru atom, which is located toward the carbene C atom, 0.3301 (5)Å from the least-squares plane of the C_20_N_4_ porphyrin core. The Ru=C bond is tilted slightly from the normal to the C_20_N_4_ least-squares plane due to steric repulsion between the porphyrinate ligand and the bulky trifluoro­methyl group. The Ru=C bond length of 1.838 (2) Å is comparable with those in bis­(subsituted phen­yl)carbene analogs.

## Related literature

For background on fluorine chemistry, see: Seebach (1990 ▶). For the preparation of the precursor of the 1-phenyl-2,2,2-trifluoro­ethyl­idene ligand, see: Shepard & Wentworth (1967 ▶). For related structures, see: Che & Huang (2002 ▶); Li *et al.* (2004 ▶); Wada *et al.* (2008 ▶). For C—H⋯π inter­actions, see: Hunter *et al.* (2001 ▶).
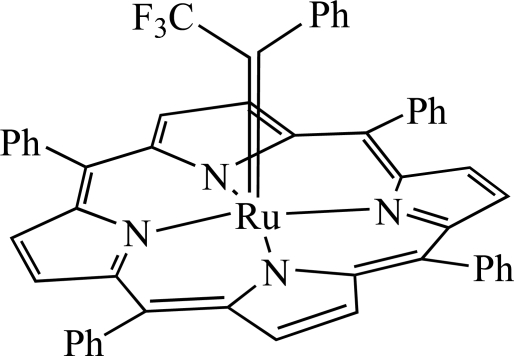


## Experimental

### 

#### Crystal data

[Ru(C_44_H_28_N_4_)(C_8_H_5_F_3_)]
*M* *_r_* = 871.89
Triclinic, 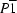

*a* = 11.131 (1) Å
*b* = 12.634 (2) Å
*c* = 15.749 (2) Å
α = 101.713 (9)°
β = 110.133 (8)°
γ = 102.15 (1)°
*V* = 1939.2 (5) Å^3^
*Z* = 2
Mo *K*α radiation
μ = 0.46 mm^−1^
*T* = 296 (2) K
0.20 × 0.20 × 0.20 mm

#### Data collection

Rigaku AFC-7R diffractometer
Absorption correction: none
9374 measured reflections
8916 independent reflections
7578 reflections with *I* > 2σ(*I*)
*R*_int_ = 0.017
3 standard reflections every 150 reflections intensity decay: 3.5%

#### Refinement

*R*[*F* ^2^ > 2σ(*F* ^2^)] = 0.031
*wR*(*F* ^2^) = 0.077
*S* = 1.03
8916 reflections
541 parameters
H-atom parameters constrained
Δρ_max_ = 0.37 e Å^−3^
Δρ_min_ = −0.46 e Å^−3^

### 

Data collection: *MSC/AFC Diffractometer Control Software* (Molecular Structure Corporation, 1993 ▶); cell refinement: *MSC/AFC Diffractometer Control Software*; data reduction: *CrystalStructure* (Rigaku/MSC, 2007 ▶); program(s) used to solve structure: *SHELXS97* (Sheldrick, 2008 ▶); program(s) used to refine structure: *SHELXL97* (Sheldrick, 2008 ▶); molecular graphics: *ORTEP-3 for Windows* (Farrugia, 1997 ▶); software used to prepare material for publication: *SHELXL97*.

## Supplementary Material

Crystal structure: contains datablocks I, global. DOI: 10.1107/S1600536808023374/lx2062sup1.cif

Structure factors: contains datablocks I. DOI: 10.1107/S1600536808023374/lx2062Isup2.hkl

Additional supplementary materials:  crystallographic information; 3D view; checkCIF report

## Figures and Tables

**Table 1:** Selected interatomic distances (Å)

H37⋯C3^i^	2.79
H37⋯C4^i^	2.72
H38⋯C1^i^	2.86

Symmetry code: (i) 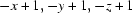.

**Fig. 1.**
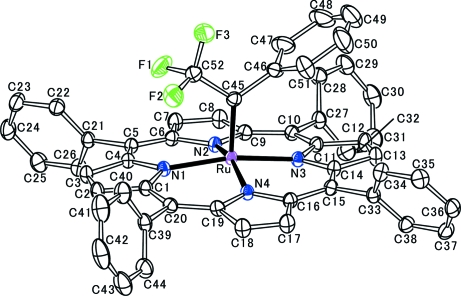
The molecular structure of (I), showing displacement ellipsoids at the 30% probability level. All H atoms have been omitted for clarity.

**Fig. 2.**
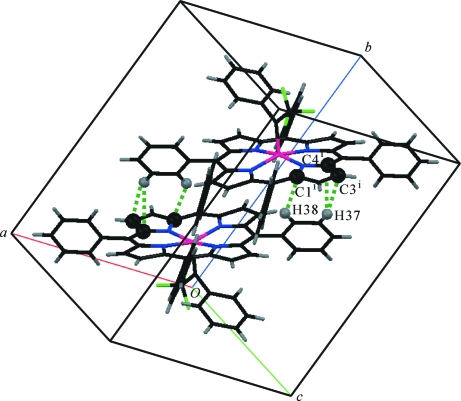
A unit-cell packing of (I). The possible CH···π interactions are drawn by dashed lines. [Symmetry code: (i) 1-x, 1-y, 1-z.]

## supplementary crystallographic information

### Comment

Ruthenium(II)-porphyrin-carbene complexes have been studied as effective and stereoselective catalysts for cyclopropanation or epoxidation of some alkenes (Che & Huang, 2002). In order to improve their robustness, activity or selectivity toward the reactions a number of compounds have been invented and applied for the reactions under different conditions (Li *et al.*, 2004; Wada *et al.*, 2008). It is important to introduce fluorine atom(*s*) for a tuning of the catalysts, taking advantage of its high electronegativity (Seebach, 1990). Surveying the carbene complexes of transition metals, few crystal structures of fluorinated alkylidene complexes have been known, especially in that fluorine atom(*s*) is attached to the β-carbon atom adjacent to the carbene carbon (α-carbon) atom. With ruthenium(II)-tetraphenylporphyrinate (tpp), we have prepared a highly stable fluorinated alkylidene complex [Ru(tpp){═C(CF_3_)Ph}] (I), and present here its X-ray structure that features the trifluoromethyl group attached to the carbene carbon atom directly.

As shown in Fig. 1, the five-coordinate ruthenium atom is bound to the carbene carbon atom to which the phenyl and trifluoromethyl groups are attached, lying in a distorted square-pyramidal geometry in (I). There seems to be no remarkable difference in the bond lengths and angles about the carbene carbon atom, compared with those for the five-coordinate ruthenium(II)-porphyrin analogs reported so far, in the ranges 1.82–1.87 Å and 111–118° (Li *et al.*, 2004; Wada *et al.*, 2008). The porphyrin core in (I) is deformed in domed conformation with maximum and minimum deviations from the C_20_N_4_ least-squares plane of 0.205 (2) and -0.209 (2) Å for N1 and C17, respectively. The trifluoromethyl group has so large van der Waals radius (2.7 Å) (Seebach, 1990) that the steric repulsion toward the porphyrin core would affect some structural features. The ruthenium atom is situated at 0.3301 (5) Å out of the C_20_N_4_ least-squares plane toward the carbene moiety in (I), while the displacements are 0.216 (2)–0.287 (1) Å in other tetraphenyl- or tetra(*p*-tolyl)porphyrin complexes (Wada *et al.*, 2008). In addition, the Ru═C45 bond is slightly tilted from the normal to the C_20_N_4_ least-squares plane, as indicated by the larger C45═Ru—N1 angle [97.70 (8)°] than the C45═Ru—N3 [93.42 (8)°]. The projection of the phenyl and trifluoromethyl groups of the carbene ligand on to the porphyrin plane shows an eclipsed configuration with regard to the Ru—N bonds.

In the unit cell a pair of the porphyrin cores of [Ru(tpp){═C(CF_3_)Ph}] are arranged facing each other across an inversion center with a distance of *ca* 4.0 Å, which is too far for the face-to-face aromatic interaction. The closer distances might suggest CH···π interactions between the porphrin core and two H atoms of the phenyl group at *meso*-position of the neighboring porphyrin (Table 1; Hunter *et al.*, 2001).

### Experimental

The carbene precursor, 1-phenyl-2,2,2-trifluorodiazoethane, was prepared according to lit. (Shepard & Wentworth, 1967). To an octane solution (50 ml) of [Ru(tpp)(CO)] (204 mg, 0.275 mmol) added an octane solution (10 ml) of 1-phenyl-2,2,2-trifluorodiazoethane (62 mg, 0.333 mmol) under nitrogen atmosphere with refluxing for 4 h. After removal of the volatiles *in vacuo*, the residue was chromatographed on a silica-gel column with dichloromethane/hexane mixture (*v*/*v*, 1/1). An intense red band was collected and evaporated to dryness. Recrystallization from a dichloromethane/hexane solution gave air-stable dark red crystals of (I) (yield, 133 mg, 0.153 mmol, 55%). Up to 450 K the compound [Ru(tpp){═C(CF_3_)Ph}] blackened and decomposed in air, but under reduced pressure (*ca* 10 Pa) it sublimed above 510 K. Spectroscopic analysis: ^1^H NMR (CDCl_3_, 500 MHz, δ, p.p.m.): 8.52 (s, 8H), 8.06–7.98 (m, 8H), 7.75–7.66 (m, 12H), 6.62 (t, J = 7.8, 1H), 6.21 (t, J = 7.8 Hz, 2H), 2.99 (d, J = 7.8 Hz, 2H), ^19^F{^1^H} NMR (CDCl_3_, 470 MHz, δ, p.p.m.): -69.1 (*s*); SIMS: m/*z* = 872 [*M*]^+^, UV/Vis (CH_2_Cl_2_): λ_max_ (log ε) 403 (5.15), 536 (3.93) nm.

### Refinement

All H atoms were placed in geometrically idealized positions and constrained to ride on their parent atoms, with C—H = 0.93 Å and *U*_iso_(H) = 1.2*U*_eq_.

### Crystal data

**Table:**

[Ru(C_44_H_28_N_4_)(C_8_H_5_F_3_)]	*Z* = 2
*M**_r_* = 871.89	*F*_000_ = 888
Triclinic, *P*1	*D*_x_ = 1.493 Mg m^−^^3^
Hall symbol: -P 1	Mo *K*α radiation λ = 0.71069 Å
*a* = 11.131 (1) Å	Cell parameters from 25 reflections
*b* = 12.634 (2) Å	θ = 14.9–15.0º
*c* = 15.749 (2) Å	µ = 0.46 mm^−^^1^
α = 101.713 (9)º	*T* = 296 (2) K
β = 110.133 (8)º	Prism, dark red
γ = 102.15 (1)º	0.20 × 0.20 × 0.20 mm
*V* = 1939.2 (5) Å^3^	

### Data collection

**Table:**

Rigaku AFC-7R diffractometer	*R*_int_ = 0.017
Radiation source: rotating Mo anode	θ_max_ = 27.5º
Monochromator: graphite	θ_min_ = 2.6º
*T* = 296(2) K	*h* = −14→0
ω/2θ scans	*k* = −16→16
Absorption correction: none	*l* = −19→20
9374 measured reflections	3 standard reflections
8916 independent reflections	every 150 reflections
7578 reflections with *I* > 2σ(*I*)	intensity decay: 3.5%

### Refinement

**Table:**

Refinement on *F*^2^	Secondary atom site location: difference Fourier map
Least-squares matrix: full	Hydrogen site location: inferred from neighbouring sites
*R*[*F*^2^ > 2σ(*F*^2^)] = 0.031	H-atom parameters constrained
*wR*(*F*^2^) = 0.077	*w* = 1/[σ^2^(*F*_o_^2^) + (0.0315*P*)^2^ + *P*] where *P* = (*F*_o_^2^ + 2*F*_c_^2^)/3
*S* = 1.03	(Δ/σ)_max_ = 0.001
8916 reflections	Δρ_max_ = 0.37 e Å^−^^3^
541 parameters	Δρ_min_ = −0.46 e Å^−^^3^
Primary atom site location: structure-invariant direct methods	Extinction correction: none

### Special details

**Table:**

Geometry. All esds (except the esd in the dihedral angle between two l.s. planes) are estimated using the full covariance matrix. The cell esds are taken into account individually in the estimation of esds in distances, angles and torsion angles; correlations between esds in cell parameters are only used when they are defined by crystal symmetry. An approximate (isotropic) treatment of cell esds is used for estimating esds involving l.s. planes.
Refinement. Refinement of F^2^ against ALL reflections. The weighted R-factor wR and goodness of fit S are based on F^2^, conventional R-factors R are based on F, with F set to zero for negative F^2^. The threshold expression of F^2^ > 2sigma(F^2^) is used only for calculating R-factors(gt) etc. and is not relevant to the choice of reflections for refinement. R-factors based on F^2^ are statistically about twice as large as those based on F, and R- factors based on ALL data will be even larger.

### Fractional atomic coordinates and isotropic or equivalent isotropic displacement parameters (Å^2^)

**Table:**

	*x*	*y*	*z*	*U*_iso_*/*U*_eq_	
Ru	0.471753 (17)	0.400275 (13)	0.271619 (12)	0.02657 (5)	
F1	0.3640 (2)	0.24901 (18)	0.04049 (12)	0.0786 (6)	
F2	0.22719 (18)	0.18331 (13)	0.09802 (13)	0.0689 (5)	
F3	0.1734 (2)	0.27779 (16)	0.00105 (12)	0.0851 (7)	
N1	0.48801 (17)	0.23935 (14)	0.24134 (12)	0.0294 (4)	
N2	0.63417 (17)	0.45653 (14)	0.24213 (12)	0.0299 (4)	
N3	0.48445 (17)	0.56603 (14)	0.32700 (12)	0.0298 (4)	
N4	0.35981 (17)	0.35415 (14)	0.34439 (12)	0.0298 (4)	
C1	0.4225 (2)	0.14717 (17)	0.26053 (15)	0.0316 (4)	
C2	0.4644 (2)	0.05090 (18)	0.23099 (17)	0.0377 (5)	
H2	0.4345	−0.0213	0.2357	0.045*	
C3	0.5547 (2)	0.08434 (18)	0.19533 (16)	0.0361 (5)	
H3	0.5985	0.0395	0.1708	0.043*	
C4	0.5711 (2)	0.20292 (17)	0.20232 (15)	0.0307 (4)	
C5	0.6656 (2)	0.27158 (17)	0.18138 (15)	0.0315 (4)	
C6	0.6940 (2)	0.38916 (18)	0.19976 (15)	0.0319 (4)	
C7	0.7891 (2)	0.45943 (19)	0.17640 (17)	0.0387 (5)	
H7	0.8423	0.4352	0.1470	0.046*	
C8	0.7876 (2)	0.56714 (19)	0.20473 (17)	0.0395 (5)	
H8	0.8404	0.6306	0.1990	0.047*	
C9	0.6906 (2)	0.56663 (17)	0.24508 (15)	0.0321 (4)	
C10	0.6577 (2)	0.66209 (17)	0.28066 (15)	0.0324 (4)	
C11	0.5596 (2)	0.66036 (17)	0.31677 (15)	0.0322 (4)	
C12	0.5197 (2)	0.75756 (18)	0.34796 (17)	0.0399 (5)	
H12	0.5536	0.8309	0.3469	0.048*	
C13	0.4246 (2)	0.72243 (18)	0.37877 (17)	0.0396 (5)	
H13	0.3797	0.7669	0.4024	0.048*	
C14	0.4047 (2)	0.60352 (17)	0.36877 (15)	0.0323 (4)	
C15	0.3193 (2)	0.53684 (18)	0.39806 (15)	0.0318 (4)	
C16	0.3048 (2)	0.42202 (18)	0.39022 (15)	0.0325 (4)	
C17	0.2271 (2)	0.3562 (2)	0.42782 (18)	0.0414 (5)	
H17	0.1823	0.3827	0.4636	0.050*	
C18	0.2304 (2)	0.2485 (2)	0.40216 (18)	0.0417 (5)	
H18	0.1872	0.1872	0.4162	0.050*	
C19	0.3125 (2)	0.24602 (18)	0.34939 (16)	0.0326 (4)	
C20	0.3387 (2)	0.14895 (17)	0.30846 (15)	0.0324 (4)	
C21	0.7482 (2)	0.21607 (17)	0.14047 (16)	0.0326 (4)	
C22	0.6894 (2)	0.1358 (2)	0.05146 (17)	0.0408 (5)	
H22	0.5974	0.1164	0.0161	0.049*	
C23	0.7674 (3)	0.0847 (2)	0.01522 (19)	0.0500 (6)	
H23	0.7273	0.0308	−0.0442	0.060*	
C24	0.9036 (3)	0.1132 (2)	0.0667 (2)	0.0520 (7)	
H24	0.9555	0.0792	0.0418	0.062*	
C25	0.9633 (3)	0.1925 (2)	0.1554 (2)	0.0489 (6)	
H25	1.0554	0.2119	0.1903	0.059*	
C26	0.8858 (2)	0.2429 (2)	0.19212 (17)	0.0400 (5)	
H26	0.9262	0.2955	0.2522	0.048*	
C27	0.7324 (2)	0.77578 (18)	0.28066 (17)	0.0356 (5)	
C28	0.7179 (3)	0.8016 (2)	0.19709 (19)	0.0444 (6)	
H28	0.6628	0.7469	0.1393	0.053*	
C29	0.7850 (3)	0.9086 (2)	0.1989 (2)	0.0539 (7)	
H29	0.7758	0.9248	0.1424	0.065*	
C30	0.8646 (3)	0.9902 (2)	0.2837 (2)	0.0583 (8)	
H30	0.9086	1.0620	0.2848	0.070*	
C31	0.8792 (3)	0.9664 (2)	0.3666 (2)	0.0583 (7)	
H31	0.9328	1.0223	0.4241	0.070*	
C32	0.8145 (3)	0.8591 (2)	0.36579 (19)	0.0481 (6)	
H32	0.8263	0.8432	0.4227	0.058*	
C33	0.2382 (2)	0.59080 (18)	0.44118 (15)	0.0323 (4)	
C34	0.0990 (2)	0.5541 (2)	0.39707 (18)	0.0408 (5)	
H34	0.0564	0.4958	0.3401	0.049*	
C35	0.0225 (2)	0.6028 (2)	0.43629 (19)	0.0460 (6)	
H35	−0.0708	0.5762	0.4063	0.055*	
C36	0.0845 (3)	0.6905 (2)	0.51967 (19)	0.0451 (6)	
H36	0.0333	0.7239	0.5458	0.054*	
C37	0.2227 (3)	0.7289 (2)	0.56426 (18)	0.0444 (6)	
H37	0.2647	0.7883	0.6206	0.053*	
C38	0.2992 (2)	0.6791 (2)	0.52544 (16)	0.0391 (5)	
H38	0.3924	0.7051	0.5562	0.047*	
C39	0.2704 (2)	0.03916 (18)	0.31815 (18)	0.0381 (5)	
C40	0.1691 (3)	−0.0434 (2)	0.2397 (2)	0.0515 (6)	
H40	0.1457	−0.0323	0.1802	0.062*	
C41	0.1022 (3)	−0.1432 (2)	0.2495 (3)	0.0689 (9)	
H41	0.0344	−0.1987	0.1965	0.083*	
C42	0.1356 (4)	−0.1602 (3)	0.3369 (3)	0.0776 (11)	
H42	0.0902	−0.2270	0.3431	0.093*	
C43	0.2352 (4)	−0.0792 (3)	0.4144 (3)	0.0702 (10)	
H43	0.2571	−0.0907	0.4737	0.084*	
C44	0.3045 (3)	0.0206 (2)	0.4057 (2)	0.0542 (7)	
H44	0.3737	0.0749	0.4589	0.065*	
C45	0.3393 (2)	0.38402 (18)	0.15662 (15)	0.0330 (4)	
C46	0.2869 (3)	0.4779 (2)	0.13103 (17)	0.0445 (6)	
C47	0.3502 (4)	0.5498 (3)	0.0933 (2)	0.0684 (9)	
H47	0.4251	0.5396	0.0832	0.082*	
C48	0.3010 (5)	0.6381 (3)	0.0703 (3)	0.0933 (14)	
H48	0.3441	0.6874	0.0458	0.112*	
C49	0.1903 (6)	0.6521 (4)	0.0837 (3)	0.1069 (17)	
H49	0.1581	0.7109	0.0683	0.128*	
C50	0.1267 (5)	0.5808 (4)	0.1193 (3)	0.1007 (14)	
H50	0.0504	0.5905	0.1273	0.121*	
C51	0.1739 (3)	0.4937 (3)	0.1439 (3)	0.0708 (9)	
H51	0.1301	0.4458	0.1690	0.085*	
C52	0.2759 (3)	0.2741 (2)	0.07449 (18)	0.0488 (6)	

### Atomic displacement parameters (Å^2^)

**Table:**

	*U*^11^	*U*^22^	*U*^33^	*U*^12^	*U*^13^	*U*^23^
Ru	0.02924 (9)	0.02307 (8)	0.02976 (9)	0.00762 (6)	0.01476 (6)	0.00812 (6)
F1	0.0846 (13)	0.1001 (15)	0.0493 (10)	0.0397 (12)	0.0318 (10)	−0.0019 (9)
F2	0.0632 (11)	0.0388 (8)	0.0758 (12)	0.0029 (8)	0.0093 (9)	0.0041 (8)
F3	0.0862 (13)	0.0716 (12)	0.0522 (10)	0.0363 (11)	−0.0189 (9)	−0.0045 (9)
N1	0.0312 (9)	0.0255 (8)	0.0343 (9)	0.0092 (7)	0.0158 (7)	0.0096 (7)
N2	0.0329 (9)	0.0259 (8)	0.0350 (9)	0.0086 (7)	0.0183 (8)	0.0095 (7)
N3	0.0331 (9)	0.0254 (8)	0.0338 (9)	0.0083 (7)	0.0175 (8)	0.0087 (7)
N4	0.0324 (9)	0.0260 (8)	0.0355 (9)	0.0094 (7)	0.0179 (8)	0.0105 (7)
C1	0.0344 (11)	0.0248 (10)	0.0360 (11)	0.0079 (8)	0.0151 (9)	0.0093 (8)
C2	0.0447 (13)	0.0232 (10)	0.0484 (13)	0.0106 (9)	0.0222 (11)	0.0113 (9)
C3	0.0416 (12)	0.0276 (10)	0.0450 (12)	0.0143 (9)	0.0229 (10)	0.0095 (9)
C4	0.0327 (10)	0.0276 (10)	0.0323 (10)	0.0105 (8)	0.0136 (9)	0.0078 (8)
C5	0.0338 (11)	0.0296 (10)	0.0338 (11)	0.0120 (8)	0.0163 (9)	0.0079 (8)
C6	0.0327 (11)	0.0317 (10)	0.0340 (11)	0.0093 (9)	0.0171 (9)	0.0098 (9)
C7	0.0414 (12)	0.0357 (11)	0.0495 (13)	0.0131 (10)	0.0293 (11)	0.0137 (10)
C8	0.0416 (12)	0.0322 (11)	0.0532 (14)	0.0082 (9)	0.0301 (11)	0.0141 (10)
C9	0.0342 (11)	0.0284 (10)	0.0361 (11)	0.0075 (8)	0.0176 (9)	0.0106 (9)
C10	0.0381 (11)	0.0271 (10)	0.0351 (11)	0.0085 (9)	0.0183 (9)	0.0110 (8)
C11	0.0386 (11)	0.0247 (10)	0.0344 (11)	0.0078 (8)	0.0175 (9)	0.0083 (8)
C12	0.0506 (14)	0.0245 (10)	0.0512 (14)	0.0107 (10)	0.0290 (12)	0.0106 (9)
C13	0.0474 (13)	0.0283 (11)	0.0514 (14)	0.0150 (10)	0.0283 (11)	0.0104 (10)
C14	0.0344 (11)	0.0274 (10)	0.0377 (11)	0.0100 (8)	0.0184 (9)	0.0076 (8)
C15	0.0314 (10)	0.0309 (10)	0.0343 (11)	0.0093 (8)	0.0160 (9)	0.0077 (8)
C16	0.0342 (11)	0.0320 (11)	0.0367 (11)	0.0111 (9)	0.0195 (9)	0.0111 (9)
C17	0.0489 (14)	0.0381 (12)	0.0531 (14)	0.0154 (10)	0.0354 (12)	0.0182 (11)
C18	0.0482 (14)	0.0349 (12)	0.0563 (15)	0.0128 (10)	0.0343 (12)	0.0196 (11)
C19	0.0331 (11)	0.0300 (10)	0.0393 (11)	0.0087 (8)	0.0188 (9)	0.0134 (9)
C20	0.0363 (11)	0.0272 (10)	0.0356 (11)	0.0079 (8)	0.0162 (9)	0.0120 (8)
C21	0.0372 (11)	0.0276 (10)	0.0392 (11)	0.0110 (9)	0.0210 (10)	0.0117 (9)
C22	0.0422 (13)	0.0399 (12)	0.0407 (12)	0.0142 (10)	0.0178 (11)	0.0093 (10)
C23	0.0616 (17)	0.0457 (14)	0.0445 (14)	0.0198 (13)	0.0267 (13)	0.0045 (11)
C24	0.0590 (16)	0.0535 (15)	0.0626 (17)	0.0301 (13)	0.0393 (15)	0.0171 (13)
C25	0.0396 (13)	0.0533 (15)	0.0624 (17)	0.0205 (12)	0.0256 (12)	0.0198 (13)
C26	0.0391 (12)	0.0368 (12)	0.0433 (13)	0.0114 (10)	0.0183 (10)	0.0075 (10)
C27	0.0404 (12)	0.0284 (10)	0.0456 (12)	0.0106 (9)	0.0249 (10)	0.0135 (9)
C28	0.0563 (15)	0.0386 (13)	0.0490 (14)	0.0159 (11)	0.0298 (12)	0.0186 (11)
C29	0.0720 (19)	0.0478 (15)	0.0694 (18)	0.0261 (14)	0.0449 (16)	0.0372 (14)
C30	0.0627 (18)	0.0360 (13)	0.093 (2)	0.0142 (13)	0.0442 (17)	0.0323 (15)
C31	0.0586 (17)	0.0326 (13)	0.0690 (19)	−0.0003 (12)	0.0201 (15)	0.0102 (12)
C32	0.0553 (15)	0.0366 (13)	0.0484 (14)	0.0045 (11)	0.0211 (12)	0.0141 (11)
C33	0.0338 (11)	0.0309 (10)	0.0382 (11)	0.0118 (9)	0.0198 (9)	0.0114 (9)
C34	0.0374 (12)	0.0358 (12)	0.0467 (13)	0.0083 (10)	0.0198 (11)	0.0054 (10)
C35	0.0333 (12)	0.0520 (15)	0.0566 (15)	0.0133 (11)	0.0226 (11)	0.0163 (12)
C36	0.0513 (14)	0.0486 (14)	0.0538 (15)	0.0249 (12)	0.0351 (13)	0.0185 (12)
C37	0.0533 (15)	0.0452 (13)	0.0389 (12)	0.0176 (11)	0.0247 (11)	0.0077 (10)
C38	0.0362 (12)	0.0424 (13)	0.0380 (12)	0.0132 (10)	0.0155 (10)	0.0086 (10)
C39	0.0440 (13)	0.0278 (10)	0.0543 (14)	0.0130 (9)	0.0296 (11)	0.0171 (10)
C40	0.0497 (15)	0.0358 (13)	0.0686 (18)	0.0077 (11)	0.0285 (14)	0.0125 (12)
C41	0.0570 (18)	0.0348 (14)	0.115 (3)	0.0056 (13)	0.0444 (19)	0.0157 (16)
C42	0.087 (2)	0.0402 (16)	0.150 (4)	0.0267 (17)	0.083 (3)	0.048 (2)
C43	0.105 (3)	0.0600 (19)	0.100 (3)	0.046 (2)	0.075 (2)	0.054 (2)
C44	0.0735 (19)	0.0410 (14)	0.0622 (17)	0.0212 (13)	0.0368 (15)	0.0242 (13)
C45	0.0350 (11)	0.0344 (11)	0.0320 (11)	0.0102 (9)	0.0159 (9)	0.0110 (9)
C46	0.0549 (15)	0.0376 (12)	0.0338 (12)	0.0156 (11)	0.0078 (11)	0.0124 (10)
C47	0.084 (2)	0.0602 (18)	0.0561 (18)	0.0131 (17)	0.0190 (16)	0.0321 (15)
C48	0.132 (4)	0.060 (2)	0.068 (2)	0.019 (2)	0.012 (2)	0.0407 (18)
C49	0.141 (4)	0.064 (2)	0.094 (3)	0.053 (3)	0.006 (3)	0.029 (2)
C50	0.100 (3)	0.088 (3)	0.122 (4)	0.066 (3)	0.030 (3)	0.035 (3)
C51	0.074 (2)	0.065 (2)	0.090 (2)	0.0413 (18)	0.0342 (19)	0.0320 (18)
C52	0.0496 (15)	0.0482 (15)	0.0396 (13)	0.0197 (12)	0.0091 (11)	0.0055 (11)

### Geometric parameters (Å, °)

**Table:**

Ru—C45	1.838 (2)	C23—H23	0.9300
Ru—N1	2.053 (2)	C24—C25	1.382 (4)
Ru—N2	2.040 (2)	C24—H24	0.9300
Ru—N3	2.053 (2)	C25—C26	1.382 (3)
Ru—N4	2.038 (2)	C25—H25	0.9300
F1—C52	1.331 (3)	C26—H26	0.9300
F2—C52	1.341 (3)	C27—C28	1.384 (3)
F3—C52	1.333 (3)	C27—C32	1.386 (3)
N1—C4	1.378 (3)	C28—C29	1.390 (3)
N1—C1	1.384 (3)	C28—H28	0.9300
N2—C9	1.385 (3)	C29—C30	1.369 (4)
N2—C6	1.388 (3)	C29—H29	0.9300
N3—C11	1.377 (3)	C30—C31	1.362 (4)
N3—C14	1.378 (3)	C30—H30	0.9300
N4—C16	1.380 (3)	C31—C32	1.390 (3)
N4—C19	1.385 (3)	C31—H31	0.9300
C1—C20	1.388 (3)	C32—H32	0.9300
C1—C2	1.439 (3)	C33—C34	1.387 (3)
C2—C3	1.346 (3)	C33—C38	1.388 (3)
C2—H2	0.9300	C34—C35	1.383 (3)
C3—C4	1.447 (3)	C34—H34	0.9300
C3—H3	0.9300	C35—C36	1.376 (4)
C4—C5	1.393 (3)	C35—H35	0.9300
C5—C6	1.398 (3)	C36—C37	1.377 (4)
C5—C21	1.499 (3)	C36—H36	0.9300
C6—C7	1.432 (3)	C37—C38	1.387 (3)
C7—C8	1.349 (3)	C37—H37	0.9300
C7—H7	0.9300	C38—H38	0.9300
C8—C9	1.428 (3)	C39—C40	1.382 (4)
C8—H8	0.9300	C39—C44	1.382 (4)
C9—C10	1.398 (3)	C40—C41	1.390 (4)
C10—C11	1.392 (3)	C40—H40	0.9300
C10—C27	1.501 (3)	C41—C42	1.370 (5)
C11—C12	1.443 (3)	C41—H41	0.9300
C12—C13	1.340 (3)	C42—C43	1.360 (5)
C12—H12	0.9300	C42—H42	0.9300
C13—C14	1.439 (3)	C43—C44	1.392 (4)
C13—H13	0.9300	C43—H43	0.9300
C14—C15	1.396 (3)	C44—H44	0.9300
C15—C16	1.400 (3)	C45—C46	1.494 (3)
C15—C33	1.495 (3)	C45—C52	1.526 (3)
C16—C17	1.428 (3)	C46—C47	1.383 (4)
C17—C18	1.350 (3)	C46—C51	1.389 (4)
C17—H17	0.9300	C47—C48	1.401 (5)
C18—C19	1.433 (3)	C47—H47	0.9300
C18—H18	0.9300	C48—C49	1.359 (6)
C19—C20	1.401 (3)	C48—H48	0.9300
C20—C39	1.499 (3)	C49—C50	1.356 (7)
C21—C22	1.390 (3)	C49—H49	0.9300
C21—C26	1.391 (3)	C50—C51	1.383 (5)
C22—C23	1.387 (3)	C50—H50	0.9300
C22—H22	0.9300	C51—H51	0.9300
C23—C24	1.376 (4)		
			
H37···C3^i^	2.79	H38···C1^i^	2.86
H37···C4^i^	2.72		
			
C45—Ru—N4	100.99 (8)	C23—C24—C25	120.0 (2)
C45—Ru—N2	98.27 (8)	C23—C24—H24	120.0
N4—Ru—N2	160.70 (7)	C25—C24—H24	120.0
C45—Ru—N1	97.70 (8)	C24—C25—C26	119.8 (2)
N4—Ru—N1	89.42 (7)	C24—C25—H25	120.1
N2—Ru—N1	89.28 (7)	C26—C25—H25	120.1
C45—Ru—N3	93.42 (9)	C25—C26—C21	120.9 (2)
N4—Ru—N3	88.58 (7)	C25—C26—H26	119.5
N2—Ru—N3	89.01 (7)	C21—C26—H26	119.5
N1—Ru—N3	168.88 (7)	C28—C27—C32	118.5 (2)
C4—N1—C1	106.29 (17)	C28—C27—C10	121.5 (2)
C4—N1—Ru	126.95 (14)	C32—C27—C10	119.9 (2)
C1—N1—Ru	126.68 (14)	C27—C28—C29	120.5 (3)
C9—N2—C6	106.62 (17)	C27—C28—H28	119.7
C9—N2—Ru	126.37 (14)	C29—C28—H28	119.7
C6—N2—Ru	126.16 (13)	C30—C29—C28	120.1 (3)
C11—N3—C14	106.12 (17)	C30—C29—H29	119.9
C11—N3—Ru	126.55 (14)	C28—C29—H29	119.9
C14—N3—Ru	126.65 (14)	C31—C30—C29	120.1 (2)
C16—N4—C19	106.40 (17)	C31—C30—H30	120.0
C16—N4—Ru	126.59 (14)	C29—C30—H30	120.0
C19—N4—Ru	126.71 (14)	C30—C31—C32	120.4 (3)
N1—C1—C20	125.66 (19)	C30—C31—H31	119.8
N1—C1—C2	109.44 (18)	C32—C31—H31	119.8
C20—C1—C2	124.67 (19)	C27—C32—C31	120.4 (3)
C3—C2—C1	107.56 (19)	C27—C32—H32	119.8
C3—C2—H2	126.2	C31—C32—H32	119.8
C1—C2—H2	126.2	C34—C33—C38	118.2 (2)
C2—C3—C4	107.30 (19)	C34—C33—C15	120.5 (2)
C2—C3—H3	126.3	C38—C33—C15	121.36 (19)
C4—C3—H3	126.3	C35—C34—C33	121.2 (2)
N1—C4—C5	125.50 (18)	C35—C34—H34	119.4
N1—C4—C3	109.39 (18)	C33—C34—H34	119.4
C5—C4—C3	124.84 (19)	C36—C35—C34	119.9 (2)
C4—C5—C6	125.03 (19)	C36—C35—H35	120.0
C4—C5—C21	117.48 (18)	C34—C35—H35	120.0
C6—C5—C21	117.42 (18)	C35—C36—C37	119.8 (2)
N2—C6—C5	126.04 (19)	C35—C36—H36	120.1
N2—C6—C7	108.92 (18)	C37—C36—H36	120.1
C5—C6—C7	125.0 (2)	C36—C37—C38	120.2 (2)
C8—C7—C6	107.54 (19)	C36—C37—H37	119.9
C8—C7—H7	126.2	C38—C37—H37	119.9
C6—C7—H7	126.2	C37—C38—C33	120.7 (2)
C7—C8—C9	108.05 (19)	C37—C38—H38	119.6
C7—C8—H8	126.0	C33—C38—H38	119.6
C9—C8—H8	126.0	C40—C39—C44	119.1 (2)
N2—C9—C10	125.95 (19)	C40—C39—C20	120.1 (2)
N2—C9—C8	108.87 (18)	C44—C39—C20	120.7 (2)
C10—C9—C8	125.18 (19)	C39—C40—C41	120.0 (3)
C11—C10—C9	124.88 (19)	C39—C40—H40	120.0
C11—C10—C27	116.57 (19)	C41—C40—H40	120.0
C9—C10—C27	118.55 (19)	C42—C41—C40	120.4 (3)
N3—C11—C10	125.57 (19)	C42—C41—H41	119.8
N3—C11—C12	109.51 (18)	C40—C41—H41	119.8
C10—C11—C12	124.92 (19)	C43—C42—C41	119.9 (3)
C13—C12—C11	107.20 (19)	C43—C42—H42	120.1
C13—C12—H12	126.4	C41—C42—H42	120.1
C11—C12—H12	126.4	C42—C43—C44	120.5 (3)
C12—C13—C14	107.6 (2)	C42—C43—H43	119.7
C12—C13—H13	126.2	C44—C43—H43	119.7
C14—C13—H13	126.2	C39—C44—C43	120.0 (3)
N3—C14—C15	125.25 (19)	C39—C44—H44	120.0
N3—C14—C13	109.39 (18)	C43—C44—H44	120.0
C15—C14—C13	125.4 (2)	C46—C45—C52	112.38 (19)
C14—C15—C16	124.24 (19)	C46—C45—Ru	124.10 (16)
C14—C15—C33	118.35 (19)	C52—C45—Ru	123.44 (17)
C16—C15—C33	117.40 (18)	C47—C46—C51	119.1 (3)
N4—C16—C15	126.30 (19)	C47—C46—C45	120.2 (3)
N4—C16—C17	109.27 (18)	C51—C46—C45	120.7 (2)
C15—C16—C17	124.4 (2)	C46—C47—C48	119.7 (4)
C18—C17—C16	107.7 (2)	C46—C47—H47	120.2
C18—C17—H17	126.1	C48—C47—H47	120.2
C16—C17—H17	126.1	C49—C48—C47	120.2 (4)
C17—C18—C19	107.39 (19)	C49—C48—H48	119.9
C17—C18—H18	126.3	C47—C48—H48	119.9
C19—C18—H18	126.3	C50—C49—C48	120.3 (4)
N4—C19—C20	125.80 (19)	C50—C49—H49	119.8
N4—C19—C18	109.13 (18)	C48—C49—H49	119.8
C20—C19—C18	125.06 (19)	C49—C50—C51	120.9 (4)
C1—C20—C19	125.15 (19)	C49—C50—H50	119.5
C1—C20—C39	118.16 (19)	C51—C50—H50	119.5
C19—C20—C39	116.69 (19)	C50—C51—C46	119.8 (4)
C22—C21—C26	118.6 (2)	C50—C51—H51	120.1
C22—C21—C5	121.1 (2)	C46—C51—H51	120.1
C26—C21—C5	120.2 (2)	F1—C52—F3	106.0 (2)
C23—C22—C21	120.3 (2)	F1—C52—F2	106.0 (2)
C23—C22—H22	119.9	F3—C52—F2	105.4 (2)
C21—C22—H22	119.9	F1—C52—C45	111.8 (2)
C24—C23—C22	120.4 (2)	F3—C52—C45	113.4 (2)
C24—C23—H23	119.8	F2—C52—C45	113.6 (2)
C22—C23—H23	119.8		
			
C45—Ru—N1—C4	89.58 (18)	C14—C15—C16—N4	−7.3 (4)
N4—Ru—N1—C4	−169.41 (17)	C33—C15—C16—N4	173.1 (2)
N2—Ru—N1—C4	−8.66 (17)	C14—C15—C16—C17	174.0 (2)
N3—Ru—N1—C4	−89.8 (4)	C33—C15—C16—C17	−5.6 (3)
C45—Ru—N1—C1	−93.94 (18)	N4—C16—C17—C18	−2.3 (3)
N4—Ru—N1—C1	7.07 (18)	C15—C16—C17—C18	176.6 (2)
N2—Ru—N1—C1	167.82 (18)	C16—C17—C18—C19	1.0 (3)
N3—Ru—N1—C1	86.7 (4)	C16—N4—C19—C20	177.2 (2)
C45—Ru—N2—C9	81.26 (18)	Ru—N4—C19—C20	3.2 (3)
N4—Ru—N2—C9	−94.9 (3)	C16—N4—C19—C18	−1.9 (2)
N1—Ru—N2—C9	178.93 (18)	Ru—N4—C19—C18	−175.98 (15)
N3—Ru—N2—C9	−12.06 (18)	C17—C18—C19—N4	0.6 (3)
C45—Ru—N2—C6	−86.67 (18)	C17—C18—C19—C20	−178.6 (2)
N4—Ru—N2—C6	97.2 (2)	N1—C1—C20—C19	−3.5 (4)
N1—Ru—N2—C6	11.00 (17)	C2—C1—C20—C19	170.4 (2)
N3—Ru—N2—C6	−179.99 (18)	N1—C1—C20—C39	176.8 (2)
C45—Ru—N3—C11	−84.89 (19)	C2—C1—C20—C39	−9.4 (3)
N4—Ru—N3—C11	174.18 (18)	N4—C19—C20—C1	3.7 (4)
N2—Ru—N3—C11	13.33 (18)	C18—C19—C20—C1	−177.3 (2)
N1—Ru—N3—C11	94.5 (4)	N4—C19—C20—C39	−176.6 (2)
C45—Ru—N3—C14	84.30 (19)	C18—C19—C20—C39	2.5 (3)
N4—Ru—N3—C14	−16.63 (18)	C4—C5—C21—C22	65.4 (3)
N2—Ru—N3—C14	−177.48 (18)	C6—C5—C21—C22	−117.6 (2)
N1—Ru—N3—C14	−96.3 (4)	C4—C5—C21—C26	−113.5 (2)
C45—Ru—N4—C16	−82.03 (19)	C6—C5—C21—C26	63.5 (3)
N2—Ru—N4—C16	94.1 (3)	C26—C21—C22—C23	−0.5 (3)
N1—Ru—N4—C16	−179.75 (18)	C5—C21—C22—C23	−179.4 (2)
N3—Ru—N4—C16	11.18 (18)	C21—C22—C23—C24	−0.4 (4)
C45—Ru—N4—C19	90.84 (19)	C22—C23—C24—C25	0.6 (4)
N2—Ru—N4—C19	−93.0 (3)	C23—C24—C25—C26	0.0 (4)
N1—Ru—N4—C19	−6.89 (18)	C24—C25—C26—C21	−0.8 (4)
N3—Ru—N4—C19	−175.96 (18)	C22—C21—C26—C25	1.0 (4)
C4—N1—C1—C20	173.6 (2)	C5—C21—C26—C25	180.0 (2)
Ru—N1—C1—C20	−3.5 (3)	C11—C10—C27—C28	−112.4 (3)
C4—N1—C1—C2	−1.1 (2)	C9—C10—C27—C28	67.6 (3)
Ru—N1—C1—C2	−178.15 (14)	C11—C10—C27—C32	65.2 (3)
N1—C1—C2—C3	0.6 (3)	C9—C10—C27—C32	−114.8 (3)
C20—C1—C2—C3	−174.1 (2)	C32—C27—C28—C29	0.4 (4)
C1—C2—C3—C4	0.2 (3)	C10—C27—C28—C29	178.0 (2)
C1—N1—C4—C5	−173.0 (2)	C27—C28—C29—C30	−1.1 (4)
Ru—N1—C4—C5	4.0 (3)	C28—C29—C30—C31	0.7 (4)
C1—N1—C4—C3	1.2 (2)	C29—C30—C31—C32	0.4 (5)
Ru—N1—C4—C3	178.23 (14)	C28—C27—C32—C31	0.7 (4)
C2—C3—C4—N1	−0.8 (3)	C10—C27—C32—C31	−177.0 (2)
C2—C3—C4—C5	173.4 (2)	C30—C31—C32—C27	−1.1 (5)
N1—C4—C5—C6	2.3 (4)	C14—C15—C33—C34	116.4 (2)
C3—C4—C5—C6	−171.1 (2)	C16—C15—C33—C34	−64.0 (3)
N1—C4—C5—C21	179.02 (19)	C14—C15—C33—C38	−63.1 (3)
C3—C4—C5—C21	5.7 (3)	C16—C15—C33—C38	116.6 (2)
C9—N2—C6—C5	−179.2 (2)	C38—C33—C34—C35	−0.9 (4)
Ru—N2—C6—C5	−9.3 (3)	C15—C33—C34—C35	179.7 (2)
C9—N2—C6—C7	0.1 (2)	C33—C34—C35—C36	1.2 (4)
Ru—N2—C6—C7	169.97 (15)	C34—C35—C36—C37	−0.6 (4)
C4—C5—C6—N2	0.6 (4)	C35—C36—C37—C38	−0.1 (4)
C21—C5—C6—N2	−176.2 (2)	C36—C37—C38—C33	0.4 (4)
C4—C5—C6—C7	−178.6 (2)	C34—C33—C38—C37	0.1 (4)
C21—C5—C6—C7	4.6 (3)	C15—C33—C38—C37	179.5 (2)
N2—C6—C7—C8	0.4 (3)	C1—C20—C39—C40	−70.2 (3)
C5—C6—C7—C8	179.7 (2)	C19—C20—C39—C40	110.1 (3)
C6—C7—C8—C9	−0.8 (3)	C1—C20—C39—C44	112.1 (3)
C6—N2—C9—C10	178.8 (2)	C19—C20—C39—C44	−67.7 (3)
Ru—N2—C9—C10	8.9 (3)	C44—C39—C40—C41	0.5 (4)
C6—N2—C9—C8	−0.6 (2)	C20—C39—C40—C41	−177.3 (2)
Ru—N2—C9—C8	−170.43 (15)	C39—C40—C41—C42	0.3 (4)
C7—C8—C9—N2	0.9 (3)	C40—C41—C42—C43	−0.3 (5)
C7—C8—C9—C10	−178.5 (2)	C41—C42—C43—C44	−0.5 (5)
N2—C9—C10—C11	−1.7 (4)	C40—C39—C44—C43	−1.3 (4)
C8—C9—C10—C11	177.6 (2)	C20—C39—C44—C43	176.5 (2)
N2—C9—C10—C27	178.2 (2)	C42—C43—C44—C39	1.4 (5)
C8—C9—C10—C27	−2.5 (3)	N4—Ru—C45—C46	93.0 (2)
C14—N3—C11—C10	177.5 (2)	N2—Ru—C45—C46	−85.7 (2)
Ru—N3—C11—C10	−11.5 (3)	N1—Ru—C45—C46	−176.11 (19)
C14—N3—C11—C12	−3.4 (2)	N3—Ru—C45—C46	3.8 (2)
Ru—N3—C11—C12	167.60 (15)	N4—Ru—C45—C52	−90.6 (2)
C9—C10—C11—N3	3.1 (4)	N2—Ru—C45—C52	90.7 (2)
C27—C10—C11—N3	−176.9 (2)	N1—Ru—C45—C52	0.3 (2)
C9—C10—C11—C12	−175.9 (2)	N3—Ru—C45—C52	−179.79 (19)
C27—C10—C11—C12	4.1 (3)	C52—C45—C46—C47	−90.1 (3)
N3—C11—C12—C13	1.6 (3)	Ru—C45—C46—C47	86.7 (3)
C10—C11—C12—C13	−179.3 (2)	C52—C45—C46—C51	89.3 (3)
C11—C12—C13—C14	0.8 (3)	Ru—C45—C46—C51	−93.9 (3)
C11—N3—C14—C15	−175.3 (2)	C51—C46—C47—C48	1.1 (5)
Ru—N3—C14—C15	13.8 (3)	C45—C46—C47—C48	−179.5 (3)
C11—N3—C14—C13	3.9 (2)	C46—C47—C48—C49	−1.0 (6)
Ru—N3—C14—C13	−167.09 (15)	C47—C48—C49—C50	0.0 (7)
C12—C13—C14—N3	−3.0 (3)	C48—C49—C50—C51	0.9 (7)
C12—C13—C14—C15	176.2 (2)	C49—C50—C51—C46	−0.8 (6)
N3—C14—C15—C16	1.1 (4)	C47—C46—C51—C50	−0.3 (5)
C13—C14—C15—C16	−177.9 (2)	C45—C46—C51—C50	−179.7 (3)
N3—C14—C15—C33	−179.2 (2)	C46—C45—C52—F1	110.7 (2)
C13—C14—C15—C33	1.7 (3)	Ru—C45—C52—F1	−66.1 (3)
C19—N4—C16—C15	−176.3 (2)	C46—C45—C52—F3	−9.1 (3)
Ru—N4—C16—C15	−2.3 (3)	Ru—C45—C52—F3	174.08 (19)
C19—N4—C16—C17	2.6 (2)	C46—C45—C52—F2	−129.5 (2)
Ru—N4—C16—C17	176.62 (15)	Ru—C45—C52—F2	53.7 (3)

Symmetry codes: (i) −*x*+1, −*y*+1, −*z*+1.
